# Rare Halophilic *Nocardiopsis* from Algerian Saharan Soils as Tools for Biotechnological Processes in Pharmaceutical Industry

**DOI:** 10.1155/2023/1061176

**Published:** 2023-05-29

**Authors:** Farida Boudjelal, Abdelghani Zitouni, Noureddine Bouras, Cathrin Spröer, Hans-Peter Klenk, Slim Smaoui, Florence Mathieu

**Affiliations:** ^1^Laboratoire de Biologie des Systèmes Microbiens (LBSM), Ecole Normale Supérieure de Kouba, Algiers, Algeria; ^2^Faculty of Biological Sciences (FSB), University of Sciences and Technologies Houari Boumediene (USTHB), BP 32 El Alia, Bab Ezzouar, 16111 Algiers, Algeria; ^3^Laboratoire de Valorisation et Conservation des Écosystèmes Arides (LVCEA), Faculté des Sciences de la Nature et de la Vie et Sciences de la Terre, Université de Ghardaia, Ghardaia, Algeria; ^4^Department Bioinformatics and Databases, Leibniz Institute DSMZ-German Collection of Microorganisms and Cell Cultures GmbH, Brunswick, Germany; ^5^School of Natural and Environmental Sciences, Newcastle University, Newcastle upon Tyne, UK; ^6^Laboratory of Microbial Biotechnology and Engineering Enzymes (LMBEE), Center of Biotechnology of Sfax (CBS), University of Sfax, Road of Sidi Mansour Km 6, P.O. Box 1177 3018 Sfax, Tunisia; ^7^Laboratoire de Génie Chimique, UMR 5503 CNRS/INPT/UPS, INP-ENSAT, 1, Université de Toulouse, Avenue de l'Agrobiopôle, 31326 Castanet-Tolosan, France

## Abstract

The Sahara Desert, one of the most extreme ecosystems in the planet, constitutes an unexplored source of microorganisms such as mycelial bacteria. In this study, we investigated the diversity of halophilic actinobacteria in soils collected from five regions of the Algerian Sahara. A total of 23 halophilic actinobacterial strains were isolated by using a humic-vitamin agar medium supplemented with 10% NaCl. The isolated halophilic strains were subjected to taxonomic analysis using a polyphasic approach, which included morphological, chemotaxonomic, physiological (numerical taxonomy), and phylogenetic analyses. The isolates showed abundant growth in CMA (complex medium agar) and TSA (tryptic soy agar) media containing 10% NaCl, and chemotaxonomic characteristics were consistent with their assignment to the genus *Nocardiopsis*. Analysis of the 16S rRNA sequence of 23 isolates showed five distinct clusters and a similarity level ranging between 98.4% and 99.8% within the *Nocardiopsis* species. Comparison of their physiological characteristics with the nearest species showed significant differences with the closely related species. Halophilic *Nocardiopsis* isolated from Algerian Sahara soil represents a distinct phyletic line suggesting a potential new species. Furthermore, the isolated strains of halophilic *Nocardiopsis* were screened for their antagonistic properties against a broad spectrum of microorganisms by the conventional agar method (agar cylinders method) and found to have the capacity to produce bioactive secondary metabolites. Except one isolate (AH37), all isolated *Nocardiopsis* showed moderate to high biological activities against *Pseudomonas syringae* and *Salmonella enterica*, and some isolates showed activities against *Agrobacterium tumefaciens*, *Serratia marcescens*, and *Klebsiella pneumoniae*. However, no isolates were active against *Bacillus subtilis*, *Aspergillus flavus*, or *Aspergillus niger*. The obtained finding implies that the unexplored extreme environments such as the Sahara contain many new bacterial species as a novel drug source for medical and industrial applications.

## 1. Introduction

Hypersaline ecosystems represent an extreme environment in which a relatively low diversity of microbial species can be found and host a particular native microflora adapted to these habitats such as halophilic bacteria [1]. The occurrence of actinobacteria in high salinity environments and tolerance of these organisms to high concentrations of salt have been previously studied by many authors, and several novel genera and species of halophilic and halotolerant have been described such as *Streptomonospora litoralis* [2], *Phytoactinopolyspora halophila* [3], *P. mesophila* [4], *Glycomyces salinus* [5], *Nesterenkonia pannonica* [6], *N. natronophila* [7], *Prauserella isguenensis* [8], *P. oleivorans* [9], *Mzabimyces algeriensis* [10], *Actinopolyspora salinaria* [11], and *Brachybacterium halotolerans* [12]. Halophiles and halotolerant actinobacteria have attracted a great attention owing to produce various bioactive natural compounds, such as antibiotics actinopolysporins A-C [13], persiamycin A [14], anticancer salternamide A [15], cytotoxic compounds nocarbenzoxazoles A-G [16], salternamides A-D [17], antiviral xiamycins C-E [18], and enzymes [19].

The Sahara is the third largest (after the cold deserts of the Antarctic and the Arctic) and hot desert in the world. The Sahara is one of the harshest environments on Earth, covering more than 9 million km^2^, spanning nearly a third of the African continent [20].

Microbial species found in the Algerian Sahara are adapted to the extreme environment characterized by high temperature and often by high salinity encountered in this area. They develop particular metabolic pathways to survive, and special phenetic properties are acquired, which can lead to a production of new substances [21].

Numerous studies have demonstrated the abundance of actinobacterial biodiversity in Algerian Saharan soil [21]. This biodiversity can lead to the discovery of novel species and secondary metabolites [22–25]. As part of this program, we focused our study on halophilic *Nocardiopsis* isolates, which were characterized using a polyphasic approach, and their antagonistic properties were evaluated against several pathogenic and nonpathogenic microorganisms. Halophilic actinobacteria are a less explored source for the discovery of novel bioactive secondary metabolites [26]. At present, seven of the thirteen validly published genera in the family *Nocardiopsaceae* [27], including *Haloactinospora*, *Lipingzhangella*, *Nocardiopsis*, *Salinactinospora*, *Spinactinospora*, *Streptomonospora*, and *Thermobifida*, contain one or several halophilic species. The genus *Nocardiopsis* currently contains 45 validly published species names among which 23 species are halophilic. The strains of *Nocardiopsis* are also known for their potential to produce bioactive metabolites [28–30].

The goal of the present study is to investigate the biodiversity of halophilic actinobacteria isolated from Algerian Saharan soils by using a polyphasic approach and to evaluate their potential to produce bioactive molecules. Actinobacteria are remarkable sources of novel antibiotics and compounds which possess medical and industrial importance.

## 2. Materials and Methods

### 2.1. Strain Isolation

Twenty-three halophilic actinobacteria were isolated by the dilution agar plating method [21], from extremely saline soil samples obtained from different regions of the Algerian Sahara. The soils in these regions are characterized by a sandy loam texture, slightly basic pH (7.5 to 8.5), and high salinity (electric conductivity at 1/5 comprised between 8 and 15 mS cm^−1^) [21].

Each dry soil sample was suspended in sterile distilled water and diluted (10 g soil in 90 mL of water). Aliquots (0.2 mL) of each dilution were spread onto a humic-vitamin agar medium [31] supplemented with NaCl (10%) and with antifungal cycloheximide (50 *μ*g/mL) to inhibit the growth of fungi. The culture plates were incubated at 30°C for two weeks, and all the colonies were directly examined by light microscopy to detect *Nocardiopsis*-like isolates. After isolation, the purified strains were stored at 4°C on agar slants of Bacto tryptic soy agar (TSA) medium supplemented with NaCl (10%) for further use.

### 2.2. Morphological Study

The cultural and morphological characteristics of selected actinobacteria were studied on yeast extract-malt extract agar (ISP 2), oatmeal agar (ISP 3), inorganic salt-starch agar (ISP 4) [32], TSA medium, and complex medium agar (CMA) [33]. All culture media were supplemented with 10% NaCl. The colors of aerial and substrate mycelia were determined with the ISCC-NBS centroid color charts [34]. The morphology of the strains grown on various media at 30°C for 12 days was examined by light microscopy for mycelia organization and sporulation.

### 2.3. Chemotaxonomic Study

For the chemotaxonomic analysis, biomass was obtained from a culture grown on a TSB medium modified with the addition of 10% NaCl and incubated at 30°C for 5 days. Analysis of diaminopimelic acid isomers and whole-cell sugar pattern was carried out using the method of Becker et al. [35] and M. Lechevalier and H. Lechevalier [36]. Phospholipids were analyzed according to the procedures developed by Minnikin et al. [37].

### 2.4. Physiological Study and Numerical Taxonomy

Fifty physiological tests were performed, including the utilization of 16 carbohydrate compounds [38]; assimilation of alanine, proline, and serine as nitrogen source; degradation of adenine, guanine, xanthine, hypoxanthine, milk casein, testosterone, Tween 80, starch, gelatin, esculin, and arbutin; decarboxylation of sodium acetate and sodium butyrate; production of nitrate reductase; growth in the presence of 0, 7, 10, 15, 20, and 25% NaCl; sensitivity to lysozyme at 0.005% and to five antibiotics; and growth at pH 5 and 9 (pH was adjusted accordingly using HCl/NaOH solutions) and at temperatures 20, 30, 37, and 42°C.

All the results of the physiological study were analyzed by numerical taxonomy. The data of phenetic characters were coded in a binary system (1/0). The degree of similarity between the studied halophilic strains was calculated by simple matching (SM), and clustering was performed by the unweighted-pair group method using average linkages (UPGMA) in the SPSS package (v.16.0.1).

### 2.5. 16S rRNA Gene Sequencing and Phylogenetic Analysis

DNA preparation was performed according to the method of Liu et al. [39]. The actinobacterial isolates were grown in a shaker (250 rpm) at 30°C for 7 days. PCR amplification of the 16S rRNA gene of the isolates was performed using two primers: 27f (5′-AGAGTTTGATCCTGGCTCAG-3′) and 1492r (5′-GTTACCTTGTTACGAC TT-3′). The sequencing reaction was performed by MilleGen Company (Toulouse, France). The same primers as before and an automated sequencer were used for this purpose. The similarities of the 16S rRNA gene sequences between strains were calculated on the basis of pairwise alignment using the EzTaxon-e server [40]. Phylogenetic and molecular evolutionary analyses were carried out using MEGA version 7.0 [41]. The 16S rRNA gene sequences of the strains were aligned using the CLUSTAL W program [42] against corresponding nucleotide sequences of *Nocardiopsis* retrieved from GenBank and EzTaxon-e. Phylogenetic trees were reconstructed by using neighbor-joining [43] with the model of Jukes and Cantor [44], maximum likelihood [45] with Kimura's two-parameter model [46], and maximum parsimony [47]. Bootstrap analysis [48] was performed to evaluate the reliability of the tree topology.

### 2.6. Antagonistic Properties

The antimicrobial spectrum was determined by the conventional agar method (agar cylinder method) as described by Patel and Brown [49] subsequently against the pathogenic and nonpathogenic fungi (*Aspergillus niger*, *A*. *flavus*, *Botrytis cinerea*, *Rhizopus nigricans*, *Saccharomyces cerevisiae* ATCC 4226, and *Kluyveromyces lactis*), Gram-positive bacteria (*Bacillus subtilis* ATCC 6633 and *Staphylococcus aureus* CIP 7625), and Gram-negative bacteria (*Agrobacterium tumefaciens* No. 2410, *Klebsiella pneumoniae* CIP 82·91, *Pseudomonas syringae*, *Salmonella enterica*, and *Serratia marcescens*). The actinobacterial isolates were grown on TSA plates supplemented with 10% NaCl for 7 days at 30°C, and then, agar cylinders (5 mm in diameter) were cut out and placed onto the agar surface (nutrient agar or Sabouraud agar, covered by 0.2 mL of culture containing 5 × 10^5^ cfu/mL for bacteria or 5 × 10^4^ cfu/mL for fungi, respectively). A sterile cylinder of TSA, supplemented with 10% NaCl, was used as control, and the plates were incubated at 30°C for 24-48 h after a diffusion process for 4 h at 4°C. The diameters of inhibition zones formed around the cylinder were then measured.

## 3. Results and Discussion

### 3.1. Cultural Characteristics and Morphology

**Figure 1:**
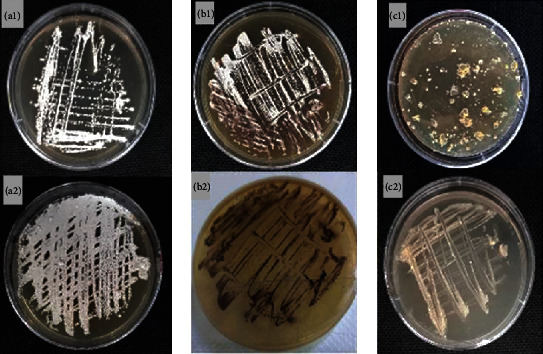
Cultural characteristics of 23 halophilic *Nocardiopsis* isolated from Saharan soils. (a_1_, a_2_) Abundant aerial mycelium of representative isolates (the same for 21 isolates). (b_1_) Abundant aerial mycelium of isolate AH25. (b_2_) Isolate AH25 with brown substrate mycelium and brown diffusible pigment. (c_1_) Separate colonies of isolate AH26 without aerial mycelium, orange substrate mycelium, and no diffusible pigment. (c_2_) Isolate AH26 without aerial mycelium, orange substrate mycelium, and no diffusible pigment. Isolates of halophilic *Nocardiopsis* grown on TSA medium supplemented with 10% NaCl for 12 days at 30°C.

All isolates have the same morphology except AH25 and AH26. The growth after 12 days at 30°C was abundant on CMA and TSA media, moderate on ISP 2 medium, and poor on ISP 4 medium. All isolates, except AH25 and AH26, produced abundant white to pale yellow aerial mycelium, a pale yellow substrate mycelium, and no diffusible pigment. Isolate AH25 produced an abundant pale pink aerial mycelium, a pink to light reddish brown substrate mycelium, and a moderate reddish brown diffusible pigment on ISP 2, CMA, and TSA media. Isolate AH26 showed only traces (microscopic) of aerial mycelium, orange substrate mycelium, and no diffusible pigment on all media tested (Table 1 and Figure 1).

All isolates produced long sporulated aerial hyphae, branched, straight to irregularly curved, often with a zigzag shape (except AH26). At the maturation state, these hyphae fragmented irregularly into long chains of elongated spores.

### 3.2. Chemical Analysis of Cellular Constituents

Cell wall hydrolysate of the 23 actinobacterial isolates contained meso-diaminopimelic acid, but glycine was not detected. Glucose and ribose were detected in whole-cell hydrolysates. Diagnostic sugars such as arabinose, madurose, xylose, or rhamnose were absent. Thus, isolates had cell walls of type III and sugar pattern type C [50]. The diagnostic phospholipids detected were phosphatidylcholine. This pattern corresponds to phospholipids type PIII [51]. Based on the morphological and chemical characteristics, isolates are attached to the genus *Nocardiopsis* described by Li et al. [52].

### 3.3. Physiological Characteristics and Numerical Taxonomy

**Figure 2:**
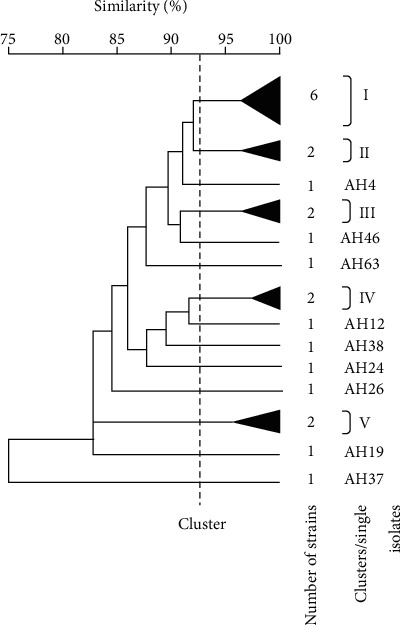
Dendrogram derived from UPGMA cluster analysis of 50 phenotypic characters, showing the relationships between the 23 halophilic *Nocardiopsis* strains isolated from Algerian Saharan soils.

The physiological characteristics based on 50 assays were subjected to numerical analysis (based on the similarity SM coefficient and UPGMA clustering). This physiological classification gave five cluster groups, designated I to V, and nine single isolates at 93.5% (Figure 2). Cluster I contains six strains, and clusters II, III, IV, and V contain two strains each. The numerical taxonomy used in this study enabled a rational distinction between isolates.

The 23 *Nocardiopsis* isolates have many similarities between them, but they also have many differences in physiological characteristics, as shown in Table 2. All isolates can grow on culture media with a NaCl concentration ranging from 7 to 15% (except AH19 which grows at 10 to 15% NaCl), and almost no isolates grow at 0% (except AH12). Some of them can tolerate up to 20% of NaCl, and optimal growth was observed at 10% for all isolates. Thus, they can be considered as moderate halophilic microorganisms, except AH12 which is halotolerant. A number of physiological characteristic differences were observed between clusters: 4 tests (clusters I-II), 5 tests (clusters I-III and III-V), 6 tests (clusters I-IV), 7 tests (clusters III-IV), and 9 tests (clusters I-V, II-III, II-IV, II-V, and IV-V). Physiological characteristic differences, sometimes more significant, were observed in the case of some single isolates such as AH26, which differs from the 22 other isolates of *Nocardiopsis* by its inability to degrade adenine, starch, D-glucose, xanthine, hypoxanthine, and Tween 80, and also several other characteristics.

### 3.4. Phylogenetic Studies

The 16S rRNA gene sequences (1529-1544 bp) of the 23 isolates were determined and deposited in the GenBank data library under the accession numbers JF777508-JF777530. The sequences were aligned with those of *Nocardiopsis* reference species available in the GenBank database, which confirmed that these 23 strains belong to the genus *Nocardiopsis*. It is interesting to note that the phylogenetic study corresponded in most cases with phenotypic taxonomy.

**Figure 3:**
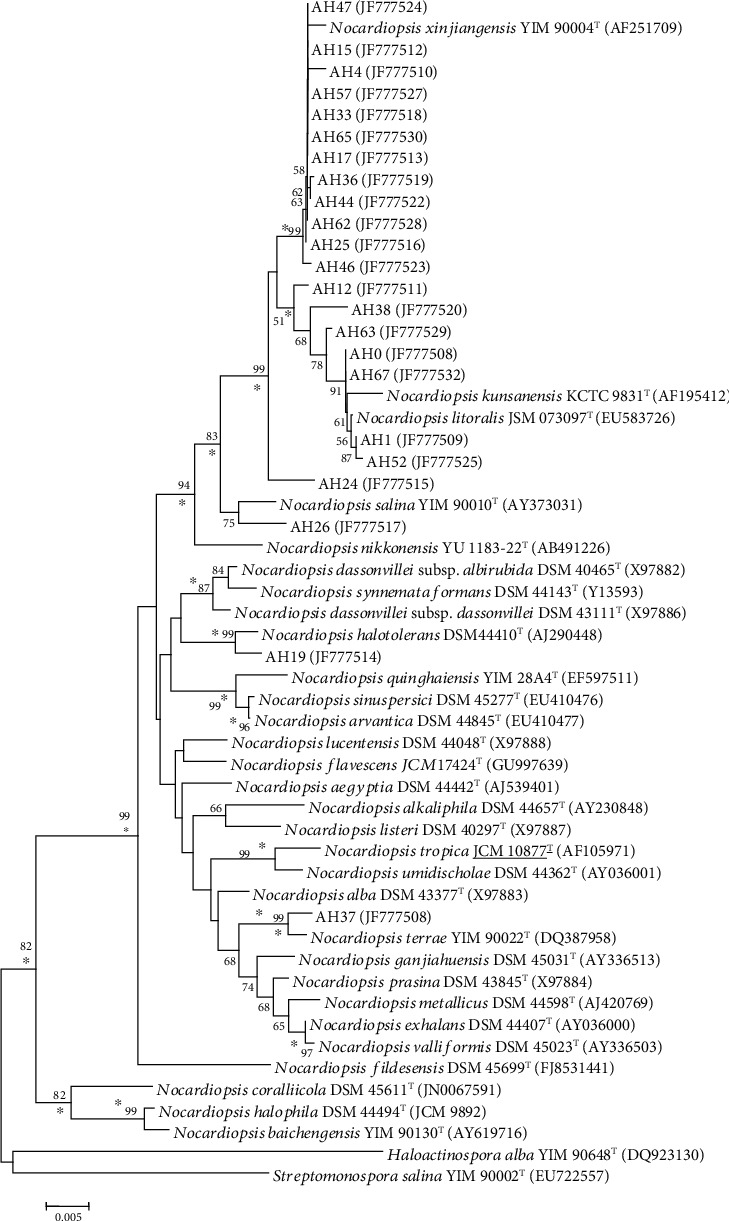
Neighbor-joining tree based on the 16S rRNA gene sequences showing the relationships between the 23 halophilic *Nocardiopsis* strains of Saharan soils and the type species of the genus *Nocardiopsis*. The accession numbers of strain sequences are given in parentheses. Asterisks indicate branches that are conserved when the neighbor-joining, maximum parsimony, and maximum likelihood methods were used in reconstructing phylogenetic trees. The numbers at the nodes indicate the levels of bootstrap support based on neighbor-joining analyses of 1000 resampled data sets; only values over 50% are given. Bar: 0.005 nucleotide substitution per nucleotide position. *Haloactinospora alba* YIM 90648^T^ and *Streptomonospora salina* YIM 90002^T^ have been used as outgroups.

The dendrogram constructed by the neighbor-joining method is shown in Figure 3. The isolates of cluster I (AH15, AH17, AH33, AH47, AH57, and AH65), cluster II (AH36 and AH44), and cluster III (AH25 and AH62), as well as the single isolate AH4, were related to *Nocardiopsis xinjiangensis* [53], with 99.4 to 99.8% 16S rRNA gene sequence similarity. The isolates of cluster IV (AH0 and AH67) were closely related to *Nocardiopsis litoralis* [54], with 99.7% similarity, followed by *N*. *kunsanensis* [55], with 99.5% similarity. The isolates of cluster V (AH1 and AH52) were also shown to be closely related to *N*. *litoralis*, but with lower percentages of similarity (99.3 to 99.6%), and more distant from *N*. *kunsanensis* (99.0 to 99.4%).

Concerning the single isolates AH38, AH19, and AH37, they exhibited relatively lower percentages of similarity, respectively, to *N*. *litoralis* (99.2%), *N*. *halotolerans* [56] (99.3%), and *N*. *terrae* [57] (99.4%). These percentages of similarity are even lower for the single isolates AH63 (98.4% with *N*. *litoralis*), AH46 (98.4% with *N*. *xinjiangensis*), AH12 (98.7% with *N*. *litoralis*), AH26 (98.8%) with *N*. *salina* [58], and AH24 (98.9% with *N*. *xinjiangensis*).

High 16S rRNA similarity values were found between representatives of validly described *Nocardiopsis* species, such as the type strains of *N*. *valliformis* and *N*. *exhalans* (99.9%) [59], *N*. *sinuspersici* and *N*. *arvandica* (99.9%) [60], *N*. *halophila* and *N*. *baichengensis* (99.9%) [61], *N*. *litoralis* and *N*. *kunsanensis* (99.6%), *N. metallicus* and *N. prasina* (99.3%), and *N*. *metallicus* [62] and *N*. *exhalans* (99.4%) [59]. It is known that strains with 16S rRNA similarity range between 98.65 and 99.99% could be a potential novel species; however, the isolates need to be the subject of more detailed molecular systematic studies based on DNA-DNA reassociation or whole-genome sequencing to determine their taxonomic status. The results were also supported by the physiological differences observed between these isolates and the nearest relatives in the genus *Nocardiopsis*. The physiological differences are given in Tables 3–5.

### 3.5. Antimicrobial Activities

The antimicrobial activities of *Nocardiopsis* isolates against several bacteria and fungi (listed in Table 6) were determined by the cylinder plate method on TSA medium supplemented with 10% NaCl.

All *Nocardiopsis* isolates (except isolate AH37) showed activities against *Pseudomonas syringae* and *Salmonella enterica* (with moderate to high activity). Some isolates showed activities against *Agrobacterium tumefaciens* (16 isolates), *Serratia marcescens* (15 isolates), and *Klebsiella pneumoniae* (16 isolates). In contrast, the activity against Gram-positive bacteria was observed only by five isolates of *Nocardiopsis* against *Staphylococcus aureus*. However, no isolates were active against *Bacillus subtilis*, *Aspergillus flavus*, or *Aspergillus niger*.

From these results, we can deduce that the halophilic actinobacteria produce molecules mainly active against Gram-negative bacteria, which appears to be interesting. There are many works reported that Gram-positive bacteria are potential biocontrol agents against Gram-negative pathogens [21–23].

High activity is obtained against *Salmonella enterica* and *Pseudomonas syringae*, which is interesting, given the toxicity and pathogenicity of *S*. *enterica* and the phytopathogenicity of *P*. *syringae*. The antifungal activity is only limited to *Kluyveromyces lactis* (16 isolates) and *Rhizopus nigricans* (12 isolates).

Up to now, *Nocardiopsis* strains and species have been shown to be the source of many secondary metabolites, such as 3-trehalosamine [63], griseusin D [64], macrolide NWA52-A [65], thiopeptide [66], nocapyrones [67], diketopiperazines [68], 4-oxo-1,4-dihydroquinoline-3-carboxamide and *N*-acetyl-anthranilic acid [69], and more recently angucyclinones [70] and kenalactams [71]. Moreover, *Nocardiopsis* strains are known to produce many novel extracellular enzymes such as amylases, inulinases, chitinases, proteases, xylanases, glucanases, and cellulases [72].

It is interesting to mention that some products derived from *Nocardiopsis* species are commercially available such as the protein kinase and NGF inhibitors K-252a [73]. The K-252a, a kinase inhibitor isolated from the culture broth of *Nocardiopsis* sp., selectively inhibits the actions of nerve growth factor (NGF) on PC12 cells (neuroendocrine tumor) [74]. An understanding on the gene clusters involved in the biosynthesis of bioactive secondary metabolites produced by many species and strains of *Nocardiopsis* would allow to increase their production by following genetic manipulation techniques and their diversity by using mutasynthetic approaches [73]. With further understanding on the biosynthetic capabilities of the several new strains and/or species, the range of molecules derived from the members of this genus and the field of “*Nocardiopsis* Biotechnology” is projected to grow in the upcoming years [73].

## 4. Conclusions

The 23 isolates were characterized by morphological characteristics and had chemotaxonomic properties consistent with their assignment to the genus *Nocardiopsis.* These isolates were compared phylogenetically and physiologically with the nearest species. The comparison showed that they are clearly different from the known species of *Nocardiopsis* and suggested the presence of a potential novel species. The antagonistic properties of the isolates showed antibacterial activity directed mainly against Gram-negative bacteria for the majority of isolates, which is interesting given the known resistance of this group of bacteria to many antibiotics. The strong activities are obtained against *Salmonella enterica*, which is interesting, given the pathogenicity and toxicity of this germ for humans. The results imply that unexplored extreme ecosystems such as the Sahara Desert potentially contain new species of actinobacteria as the source of novel bioactive secondary metabolites that may serve as a structural foundation for the development of novel drugs to be used in medicine and/or other industries.

## Figures and Tables

**Table 1:** Cultural characteristics of 23 halophilic *Nocardiopsis* isolated from Saharan soils.

Medium with 10% NaCl	Group	Number of isolates	Growth	Color of aerial mycelium	Color of substrate mycelium	Diffusible pigment
Yeast extract-malt extract agar (ISP2)	G1	21	++	White to pale yellow	Pale yellow	None
	G2	01	++	Pale pink	Light reddish brown	Moderate reddish brown
	G3	01	++	None	Orange	None

Inorganic salt-starch agar (ISP4)	G1	21	+	Yellowish	Moderate yellow	None
	G2	01	+	None	Moderate reddish pink	None
	G3	01	+	None	None	None

Bacto tryptic-soy agar (TSA)	G1	21	+++	Light yellow	Pale yellow	None
	G2	01	+++	Pale pink	Light reddish brown	Moderate reddish brown
	G3	01	+++	None	Orange	None

Complex medium agar (CMA)	G1	21	+++	Light yellow	Pale yellow	None
	G2	01	+++	Pale pink	Light reddish brown	Moderate reddish brown
	G3	01	+++	None	Orange	None

+++ = abundant; ++ = moderate; + = poor. All media were supplemented with 10% (*w*/*v*) NaCl (pH 7.2). Colors were taken from ISCC-NBS color charts [32].

**Table 2:** Physiological characteristics of *Nocardiopsis* isolates.

Properties	Clusters and isolates					Single isolates								
	I	II	III	IV	V									
	AH15, AH17, AH33, AH47, AH57, and AH65	AH36, AH44	AH25, AH62	AH0, AH67	AH1, AH52	AH4	AH12	AH19	AH24	AH26	AH37	AH38	AH46	AH63
Degradation of														
Arbutin	+	+	+	–	V	–	–	–	+	+	+	–	+	+
Casein	+	–	+	+	–	+	+	+	+	–	+	–	–	+
Esculin	+	+	+	–	+	–	–	–	+	+	+	–	+	+
Carbone source utilization:														
Adonitol	+	v	–	+	V	–	+	+	+	–	–	+	–	+
Cellobiose	+	+	+	+	+	+	+	+	–	–	+	+	+	+
Fructose	+	+	+	–	–	–	+	+	–	–	+	+	+	+
Galactose	+	+	+	+	–	–	–	+	+	–	+	+	+	+
Inositol	V	+	–	–	–	–	–	+	–	–	+	+	+	–
Lactose	V	+	V	–	V	+	–	+	–	+	+	+	–	+
Mannitol	+	–	+	+	–	+	–	+	+	+	+	+	–	+
Mannose	+	+	–	v	–	–	+	+	–	–	+	+	+	+
Melibiose	–	+	–	–	–	–	–	–	–	+	+	–	–	–
Raffinose	–	–	–	–	–	–	–	–	–	+	–	+	–	–
Rhamnose	+	+	–	+	–	–	+	+	+	+	–	+	–	–
Ribose	+	+	+	+	+	+	+	+	–	+	+	–	–	+
Sucrose	–	–	+	+	–	+	+	+	+	–	–	–	–	+
Xylose	+	+	+	+	+	–	–	+	+	–	+	+	–	+
Nitrogen source utilization:														
Alanine	+	+	+	+	–	+	+	+	–	+	+	+	+	+
Proline	+	+	+	+	+	–	+	+	+	+	–	+	+	+
Decarboxylation of sodium acetate	–	+	–	+	–	–	–	+	+	–	+	+	–	+
Nitrate reduction	+	+	+	+	+	+	+	+	+	+	+	–	+	+
Growth in the presence of														
NaCl 0% *w*/*v*	–	–	–	–	–	–	+	–	–	–	–	–	–	–
NaCl 20% *w*/*v*	–	–	+	–	+	–	–	–	+	–	–	–	+	–
Lysozyme 0.005% *w*/*v*	–	V	V	+	+	+	+	+	–	+	–	–	–	+
Growth at 42°C	–	–	–	–	–	–	+	–	–	+	–	–	–	+

+, positive; –, negative; V, variable. All experiments were done in duplicate.

**(a):** 

Characteristics	Isolates AH													*N. xinjiangensis* YIM 90004^T^
	4	15	17	24	25	33	36	44	46	47	57	62	65	
Degradation of organic compounds:														
Starch	+	+	+	+	+	+	+	+	+	+	+	+	+	-
Gelatin	+	+	+	+	+	+	+	+	+	+	+	+	+	-
Carbon source utilization:														
Cellobiose	+	+	+	-	+	+	+	+	+	+	+	+	+	+
Fructose	-	-	+	-	+	+	+	+	+	+	+	+	+	+
Galactose	-	+	+	+	+	+	+	+	+	+	+	+	+	+
Glucose	+	+	+	+	+	+	+	+	+	+	+	+	+	-
Melibiose	-	+	-	-	-	+	+	-	-	+	+	-	+	-
Raffinose	-	-	-	+	-	+	-	-	-	-	+	-	-	-
Ribose	+	+	+	-	-	+	+	+	-	+	+	+	+	-
Xylose	-	+	+	+	+	+	+	+	-	-	+	+	+	-
Nitrogen source utilization:														
Alanine	+	-	+	+	+	+	+	+	+	+	+	+	+	+
Proline	-	+	+	+	+	+	+	+	+	-	+	+	+	+
Serine	-	+	+	-	-	+	+	+	+	+	-	-	-	+
Nitrate reductase	+	+	+	+	+	+	+	+	+	+	+	+	-	-
Decarboxylation of sodium salts:														
Acetate	-	+	-	+	+	-	+	+	+	+	-	+	+	-

**(b):** 

Temperature range (optimum) (°C)	28-37 (30)	20-40 (28)
pH range (optimum)	7-9 (7)	6.0-10.0 (7.2)
Diagnostic sugars	Glu, rib	Xylose, arabinose, and galactose
Major phospholipids	PG, PC, PME, and PI	PG, PI

+, property present; -, property absent. All experiments were done in duplicates. DPG: diphosphatidylglycerol; PC: phosphatidylcholine; PE: phosphatidylethanolamine; PG: phosphatidylglycerol; PI: phosphatidylinositol; PME: phosphatidylmethylethanolamine.

**(a):** 

Characteristics	Isolates AH								*N. litoralis* JSM 073097^T^	*N. kunsanensis* HA-9^T^
	0	1	12	38	52	63	64	67		
Degradation of organic compounds:										
Casein	+	+	+	-	-	+	+	+	-	+
Esculin	+	-	-	-	+	+	-	-	-	-
Starch	+	+	+	+	+	+	+	+	-	+
Tween 80	+	+	+	+	+	+	+	+	-	
Carbon source utilization:										ND
Adonitol	+	+	+	+	-	+	+	+	-	ND
Arabinose	+	+	-	+	+	+	+	+	-	ND
Cellobiose	+	+	+	+	+	+	+	+	-	-
Fructose	+	-	+	+	+	+	-	+	-	+
Galactose	+	-	-	+	+	+	+	-	-	-
lactose	-	-	-	+	+	+	+	+	-	
Maltose	+	+	+	+	+	+	+	+	-	-
Mannitol	+	-	-	+	+	+	+	+	-	-
Melibiose	-	-	-	-	+	+	-	-	-	ND
Raffinose	-	-	-	+	+	+	-	-	-	-
Rhamnose	+	-	+	+	+	+	-	+	-	-
Ribose	+	+	+	-	+	+	+	+	-	ND
Trehalose	-	+	-	-	+	+	+	+	-	-
Xylose	+	+	-	+	+	+	+	-	+	-
Nitrogen source utilization:										
Alanine	+	-	+	+	+	+	+	+	+	+
Proline	+	+	+	+	+	+	+	+	-	-
Serine	-	+	+	+	+	+	+	+	-	-
Nitrate reductase	+	+	+	-	+	+	+	+	-	-
Decarboxylation of sodium salts:										
Acetate	+	+	-	+	+	+	+	+	-	-
Citrate	+	+	+	+	+	+	+	+	-	ND

**(b):** 

NaCl range (optimum) (%; *w*/*v*)			
Temperature range (optimum) (°C)	28-37 (30)	20-35 (25)	28-37 (30)
pH range (optimum)	7-9 (7)	6.0-10.5 (8.5)	7.0-11.0 (9.0)
Diagnostic sugars	Glu, rib	None	None
Major phospholipids	PG, PC, PME, and PI	DPG, PC, and PG	DPG, PC, and PG

+, property present; -, property absent; ND: not determined. All experiments were done in duplicates. DPG: diphosphatidylglycerol; PC: phosphatidylcholine; PE: phosphatidylethanolamine; PG: phosphatidylglycerol; PI: phosphatidylinositol; PME: phosphatidylmethylethanolamine.

**Table 5:** Characteristics distinguishing strain AH37 from related species *Nocardiopsis terrae.*

Characteristics	AH37	*Nocardiopsis terrae* YIM 90022^T^
Aerial mycelium color	Pale yellow	White
Diffusible pigment on ISP2	None	Deep brown
Utilization of organic compounds:		
Esculin	+	-
Gelatin	+	-
Tween 80	+	-
Growth on sole carbon source (1%, *w*/*v*):		
Arabinose	+	-
Galactose	+	-
Lactose	+	-
Mannitol	+	-
Maltose	+	-
Melibiose	+	-
Acetate	+	-
Proline	+	-
Growth in the presence of NaCl % (*w*/*v*)		
0	-	-
3	-	+++
5	-	+++
7	+++	+
10	++	+
15	++	+
NaCl range optimum (%; *w*/*v*)	7-15 (7)	1-15 (3-5)
pH range optimum	7-9 (7)	6.0-10,5 (8.5)
Temperature range (optimum) (°C)	28-37 (30)	10-45 (30)
Major phospholipids	PC, PG, PME, and PI	DPG, PC, PG, and PME

+, property present; -, property absent. All experiments were done in duplicates. DPG: diphosphatidylglycerol; PC: phosphatidylcholine; PG: phosphatidylglycerol; PI: phosphatidylinositol; PME: phosphatidylmethylethanolamine.

**Table 6:** Antimicrobial activities of halophilic *Nocardiopsis* isolates against various microorganisms.

Bioassay organisms	Clusters and isolates					Single isolates								
	I	II	III	IV	V									
	AH15, AH17, AH33, AH47, AH57, and AH65	AH36, AH44	AH25, AH62	AH0, AH67	AH1, AH52	AH4	AH12	AH19	AH24	AH26	AH37	AH38	AH46	AH63
*Staphylococcus aureus*	–	10 to 12	–	–	–	–	–	13	12	–	–	–	11	–
*Agrobacterium tumefaciens*	10 to 15	10 to 14	11 to 13	–	10 to 19	11	11	13	11	11	–	–	–	–
*Klebsiella pneumoniae*	–	10 to 14	–	–	10 to 16	13	–	11	–	–	–	–	14	–
*Pseudomonas syringae*	12 to 24	18 to 20	13 to 16	12 to 22	12 to 20	24	11	23	12	18	–	18	20	12
*Salmonella enterica*	16 to 20	15 to 20	10 to 20	15 to 25	14 to 22	12	15	20	25	20	–	18	21	17
*Serratia marcescens*	10 to 20	10 to 15	11 to 18	18 to 25	–	12	–	20	20	–	–	–	–	11
*Kluyveromyces lactis*	10 to 13	16 to 30	10 to 17	–	–	12	13	11	20	15	–	14	–	15
*Rhizopus nigricans*	20 to 30	15 to 30	10 to 30	–	10 to 28	–	–	–	20	–	–	–	20	–

–, no activity. The numbers indicated the diameters of inhibition zones (in millimeter) using the agar cylinder method. The diameter of the cylinder agar (5 mm) does not include in the measurements. Agar cylinders (not seeded with actinomycetes) were used as controls.

## Data Availability

The data used to support the findings of this study are included within the article. Raw data are available from the corresponding author upon request.
